# Insights into the genetic and host adaptability of emerging porcine circovirus 3

**DOI:** 10.1080/21505594.2018.1492863

**Published:** 2018-08-24

**Authors:** Gairu Li, Huijuan Wang, Shilei Wang, Gang Xing, Cheng Zhang, Wenyan Zhang, Jie Liu, Junyan Zhang, Shuo Su, Jiyong Zhou

**Affiliations:** a MOE Joint International Research Laboratory of Animal Health and Food Safety, Institute of Immunology, Nanjing Agricultural University, Nanjing, China; b Jiangsu Engineering Laboratory of Animal Immunology, College of Veterinary Medicine, Nanjing Agricultural University, Nanjing, China; c Key laboratory of Animal Virology of Ministry of Agriculture, Zhejiang University, Hangzhou, China

**Keywords:** Porcine circovirus 3 (PCV3), codon usage bias, natural selection, dinucleotide, host

## Abstract

Porcine circovirus 3 (PCV3) was found to be associated with reproductive disease in pigs, and since its first identification in the United States, it subsequently spread worldwide, especially in China, where it might pose a potential threat to the porcine industry. However, no exhaustive analysis was performed to understand its evolution in the prospect of codon usage pattern. Here, we performed a deep codon usage analysis of PCV3. PCV3 sequences were classified into two clades: PCV3a and PCV3b, confirmed by principal component analysis. Additionally, the degree of codon usage bias of PCV3 was slightly low as inferred from the analysis of the effective number of codons. The codon usage pattern was mainly affected by natural selection, but there was a co-effect of mutation pressure and dinucleotide frequency. Moreover, based on similarity index analysis, codon adaptation index analysis and relative codon deoptimization index analysis, we found that PCV3 might pose a potential risk to public health though with unknow pathogenicity. In conclusion, this work reinforces the systematic understanding of the evolution of PCV3, which was reflected by the codon usage patterns and fitness of this novel emergent virus.

## Introduction

Circovirus belongs to the Circoviridae. It is a small monomeric single-stranded circular DNA virus with a genome size of approximately 2 kb. Circoviruses can transmit among birds, pigs, dogs, fish, mink, bats and foxes [1–6]. Only two circovirus species, porcine circovirus type 1 and 2 (PCV1 and PCV2), were reported in pigs before 2015. PCV1 does not appear to cause clinical disease in pigs. However, PCV2 infection is known to cause multiple clinical signs and poses a serious threat to the pig industry worldwide [1,7].

In 2015, a novel porcine circovirus 3 (PCV3) was first reported in the USA by metagenomic analysis. It is a genetically divergent circovirus associated with porcine dermatitis and nephropathy syndrome (PDNS). Similar to PCV2, the PCV3 genome harbours two major open reading frames (ORFs). ORF1 encodes the replication-associated protein (Rep) and ORF2 encodes the capsid (Cap) protein [8]. It is noteworthy that in less than two years there were extensive reports on the detection of PCV3 in many countries including the USA, China, Brazil, Italy, Korea, Thailand, Spain, Denmark, Germany, Sweden and Poland [9–17]. Though the evolution of PCV3 has been reported in previous studies [12,18,19], the standard methods in exploring the genotyping identification still controversial and the pathogenicity of PCV3 was unclear [13,20], which needs further research.

Phylogenetic analysis is known to be a powerful tool to investigate virus evolution [21]. However, codon usage bias analysis provides a different perspective regarding virus evolution. Several studies have documented the species-specific phenomenon of codon usage bias [22–25], which refers to the preferential use of certain synonymous codons [26]. Studies on codon usage have identified several factors that can influence codon usage patterns. These include mutation pressure, natural or translational selection, secondary protein structure, replication, selective transcription, hydrophobicity and hydrophilicity of the protein and the external environment [27–31]. When the size of the viral genome and other viral features, such as its dependence on the host machinery for key processes (including replication, protein synthesis and transmission) are compared to those of prokaryotic and eukaryotic genomes, the interplay between the codon usage of the virus and that of its host is expected to affect the overall viral survival, fitness, evasion of the host immune system and evolution [29,32]. Previous studies showed that the codon usage bias of PCV was low, while mutation pressure plays a key role in shaping the codon usage bias of PCV1, mutation pressure and natural selection contribute equally to the codon usage bias of PCV2 [33].

Here, we performed a detailed study of the evolutionary processes reflected by codon usage pattern of emerging PCV3. The combination of codon usage bias and traditional phylogenetic analyses of PCV3 coding sequences provides a novel perspective of the genetic divergence of emerging PCV3 and possibly supports the idea of an ongoing genotype shift.

## Results

### Recombination and phylogenetic analysis

Recombination events can mislead evolution analysis [34] as well as codon usage analysis [35]. Therefore, we looked for potential recombination events. No recombination events were observed. However, the China/GD2016 (KY418606) strain was excluded from the analysis because of low quality. Therefore, a total of 51 strains were analysed.

Before the codon usage analysis of different clades of PCV3, PCV3 NJ trees using 51 strains were inferred. Phylogenetic tree (Figure S1) revealed that two stable clusters, 3a and 3b, were identified, which was named by our previous study [36]. Furthermore, 3a clade could be divided into two stable individual subclades, 3a-1 and 3a-2, and immediate clade (IM), due to the instable distribution. The observed result have also been very similar general topologies in our previous study focus on researching the genotype identification of PCV3 [36].

### PCA analysis

**Figure 1.**
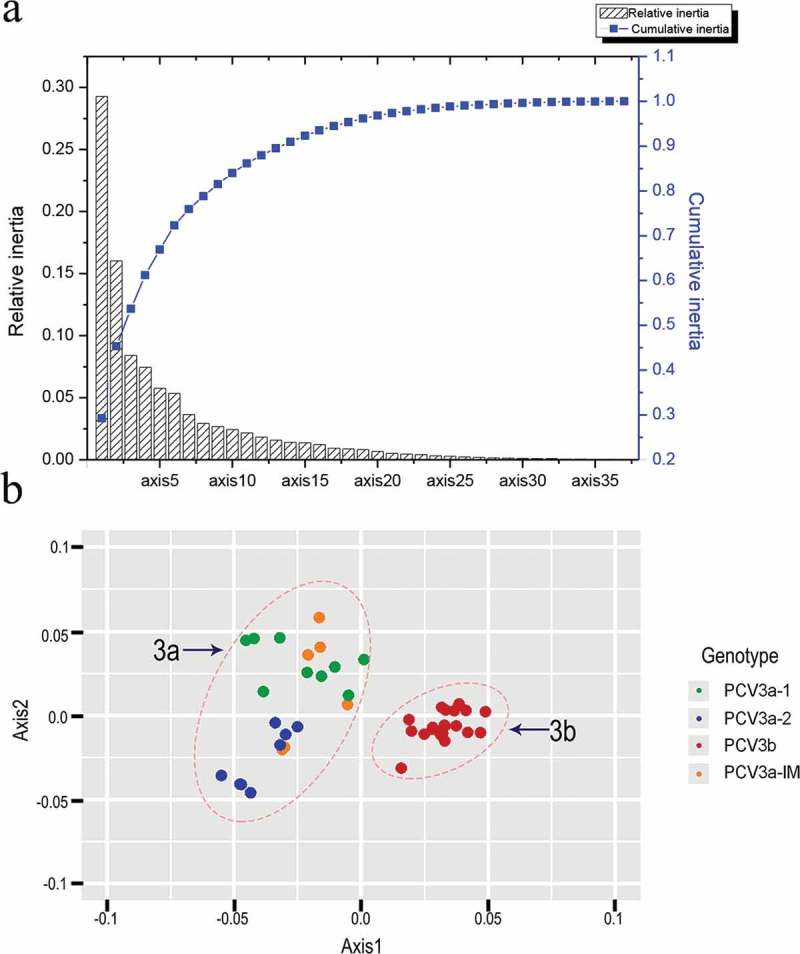
(a) The relative and cumulative inertia of the first 35 axes from a COA of the RSCU values; (b) PCA of different genotypes. Green, blue, red and orange refer to PCV3a-1, PCV3a-2, PCV3a-IM and PCV3b, respectively.

PCA analysis (axis1 plotted against axis2) is a widely used multivariate statistical approach [36] to identify the major trends in codon usage variation among genes. It involves the distribution of 59 synonymous codons in 59 dimensions. The values of the first four axes were 29.29%, 16.03%, 8.4% and 7.5% (Figure 1(a)) revealing that axis1 was the major factor affecting codon usage. Next, axis 1 was plotted against axis 2. We found that points were divided into two groups (3a, 3b) (Figure 1(b)). In addition, PCV3a-1 and PCV3a-2 clustered separately and PCV3a-IM among them. The PCV3a-1, PCV3a-2 and PCV3a-IM clades were all part of group 3a, while PCV3b all grouped together, consistent with the phylogenetic clustering.

### Nucleotide composition analysis

Next, we explored if the nucleotide composition has an influence on codon usage bias. The average ± standard deviation (SD) values of nucleotides A and G were 28.31% ± 0.11 and 26.09% ± 0.12, respectively, and more abundant than C (22.47% ± 0.11) and T (23.13% ± 0.10). However, the nucleotide composition at the third position of synonymous codons (A_3_, C_3_, G_3_ and T_3_) were significantly different from the nucleotide composition. The most frequent nucleotide was T_3_ (34.36% ± 0.003), followed by G_3_ (32.70% ± 0.004), C_3_ (29.06% ± 0.003) and A_3_ (28.53% ± 0.004). Additionally, the percentage of AT (51.4% ± 0.002) was higher than GC (48.6% ± 0.002) revealing that PCV3 strains are AT rich. The average values of GC at the first, second and third positions (GC_12s_, GC_3s_) were 48.55% ± 0.001, 48.57% ± 0.004, respectively. In addition, the nucleotide compositions of the different genotypes (PCV3a-1, PCV3a-2, PCV3a-IM and PCV3b) were similar to the combined strains (Table S2).

### PCV3 coding sequences have a low codon usage bias

**Figure 2.**
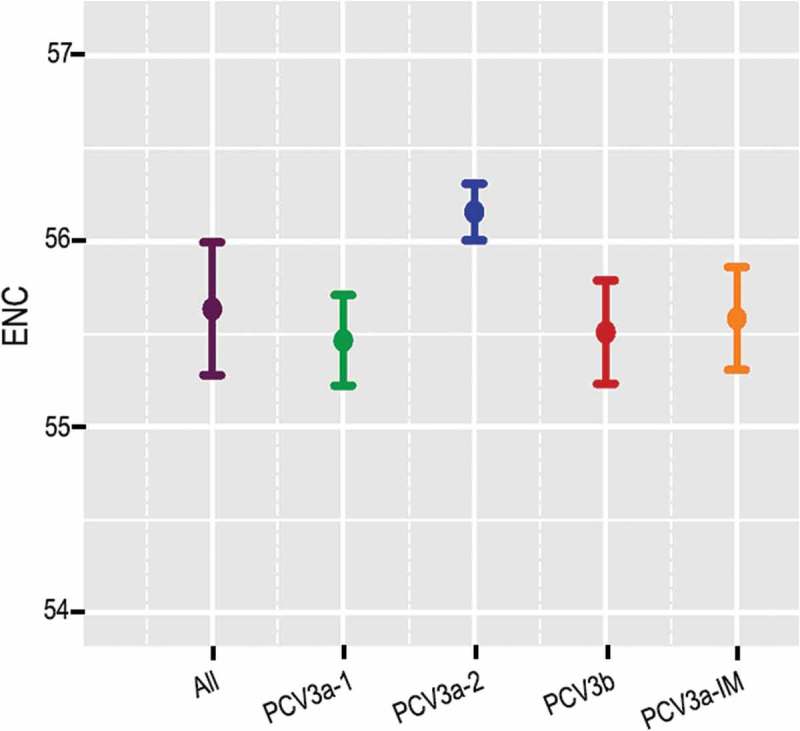
ENC values of PCV3 and the different genotypes. Green, blue, red and orange represented PCV3a-1, PCV3a-2, PCV3a-IM and PCV3b, respectively.

The ENC value was estimated to evaluate the extent of codon usage bias of PCV3 in relation to all the strains and individual genotypes. The ENC value of all the strains ranged from 54.89 to 56.3 with mean of 55.52 (SD ± 0.26), indicating low codon usage bias. Additionally, the mean values of the different genotypes were 55.469 (SD ± 0.24), 56.159 (SD ± 0.15), 55.585 (SD ± 0.28) and 55.513 (SD ± 0.28) for PCV3a-1, PCV3a-2, PCV3a-IM and PCV3b, respectively (Figure 2) suggesting low codon usage bias.

### Relative synonymous codon usage (RSCU) analysis

The RSCU was calculated for whole sequences, different PCV3 genotypes and potential hosts. Among the 18 frequently used synonymous codons, 11 were A/T-ended codons, 7 were T-ended, followed by A-, C- and G-ended codons (Table 1). This indicates that PCV3 has a preference for A/T-ended compared to G/C-ended codons. Regarding over-and under-represented synonymous codons of optimal synonymous codons, 5, including His (CAC), Ile (ATT), Ser (AGC), Val (GTT) and Arg (AGA), had RSCU values > 1.6 and none of them was under-represented (RSCU value < 0.6). However, there were no significant differences among the different PCV3 genotypes, except that 2 preferred synonymous codons of PCV3b encoding for Ala and Arg were GCT and AGG, respectively, differing from other genotypes which used GCG for Ala and AGA for Arg. Furthermore, to determine the influence of the host species on the synonymous codon usage pattern of PCV3, the RSCU values of *Sus scrofa, Homo sapiens, Canis familiaris* and *Rhinolophus ferrumequinum* were determined. We found that only 5 of 18 abundant codons, including His (CAC), Asn (AAC), Glu (GAG), Ser (AGC) and Thr (ACC), were identical when analysed as a whole or as each genotype.

**Table 1.** RSCU analysis of PCV3 genotypes and potential hosts.

			Genotype				Potential host			
AA	Codon	PCV3	PCV3a-1	PCV3a-2	PCV3a-IM	PCV3b	*Rhinolophus ferrumequinum*	*Sus scrofa*	*Homo sapiens*	*Canis familiaris*
Ala	GCA	0.62	0.68	0.69	0.69	0.55	0.64	0.74	0.91	0.79
	GCC	0.93	0.97	0.97	0.9	0.89	***2.32***	***1.8***	***1.6***	***1.75***
	GCG	1.21	1.14	1.1	1.11	**1.31**	0.46	0.5	0.42	0.46
	GCT	**1.24**	**1.2**	**1.24**	**1.29**	1.25	0.58	0.96	1.06	1
Cys	TGC	0.85	0.79	0.86	0.86	0.85	**1.20**	**1.21**	**1.09**	**1.15**
	TGT	**1.15**	**1.21**	**1.14**	**1.14**	**1.15**	0.80	0.79	0.91	0.85
Asp	GAC	0.77	0.78	0.78	0.76	0.77	**1.25**	1.2	1.07	1.14
	GAT	**1.23**	**1.22**	**1.22**	**1.24**	**1.23**	0.75	0.8	0.93	0.86
Glu	GAA	0.90	0.87	0.89	0.91	0.91	0.63	0.72	0.84	0.79
	GAG	**1.10**	**1.13**	**1.11**	**1.09**	**1.09**	**1.37**	**1.28**	**1.16**	**1.21**
Phe	TTC	0.92	0.91	0.92	0.92	0.93	**1.05**	**1.21**	**1.07**	**1.18**
	TTT	**1.08**	**1.09**	**1.08**	**1.08**	**1.07**	0.95	0.79	0.93	0.82
Gly	GGA	1.00	1.04	1.04	1.06	0.95	0.93	0.91	1	0.97
	GGC	0.57	0.57	0.56	0.57	0.58	***1.94***	**1.46**	**1.35**	**1.39**
	GGG	**1.53**	**1.56**	**1.53**	**1.54**	**1.52**	0.51	1.05	1	1
	GGT	0.90	0.83	0.87	0.83	0.96	0.63	0.57	0.65	0.65
His	CAC	***1.82***	***1.82***	***1.82***	***1.82***	***1.82***	**1.22**	**1.3**	**1.16**	**1.22**
	CAT	0.18	0.18	0.18	0.18	0.18	0.78	0.7	0.84	0.78
Ile	ATA	0.87	0.85	1	0.83	0.85	0.10	0.42	0.51	0.45
	ATC	0.14	0.12	0.2	0.16	0.12	***1.94***	***1.67***	**1.41**	***1.6***
	ATT	***1.98***	***2.03***	***1.8***	***2.01***	***2.03***	0.97	0.91	1.08	0.96
Lys	AAA	**1.16**	**1.16**	**1.16**	**1.17**	**1.17**	0.63	0.76	0.87	0.79
	AAG	0.84	0.84	0.84	0.83	0.83	**1.37**	**1.24**	**1.13**	**1.21**
Leu	CTA	0.76	0.8	0.75	0.75	0.76	0.27	0.58	0.43	0.39
	CTC	1.30	1.31	1.27	1.31	1.3	1.38	1.07	1.17	1.3
	CTG	1.44	1.29	1.44	1.47	1.5	***2.60***	***2.4***	***2.37***	***2.56***
	CTT	0.59	0.59	0.71	0.56	0.55	0.64	0.77	0.79	0.7
	TTA	0.38	0.37	0.36	0.41	0.38	0.42	0.38	0.46	0.35
	TTG	**1.53**	***1.64***	**1.47**	**1.5**	**1.51**	0.69	0.79	0.77	0.71
Asn	AAC	**1.14**	**1.06**	**1.17**	**1.05**	**1.18**	**1.37**	**1.21**	**1.06**	**1.13**
	AAT	0.86	0.94	0.83	0.95	0.82	0.63	0.79	0.94	0.87
Pro	CCA	**1.21**	**1.3**	**1.16**	**1.23**	**1.19**	0.88	0.94	1.11	1.01
	CCC	0.90	0.89	0.9	0.9	0.9	**1.56**	**1.46**	**1.29**	**1.42**
	CCG	0.85	0.79	0.89	0.84	0.86	0.44	0.56	0.45	0.48
	CCT	1.04	1.02	1.05	1.03	1.04	1.13	1.05	1.15	1.08
Gln	CAA	**1.33**	**1.33**	**1.33**	**1.33**	**1.32**	0.29	0.44	0.53	0.5
	CAG	0.67	0.67	0.67	0.67	0.68	***1.71***	**1.56**	**1.47**	**1.5**
Arg	AGA	***1.66***	***1.73***	***1.75***	***1.68***	1.58	***1.70***	1.12	**1.29**	1.19
	AGG	1.55	1.51	1.49	1.52	**1.59**	1.20	1.23	1.27	**1.27**
	CGA	0.38	0.39	0.39	0.36	0.38	0.60	0.6	0.65	0.64
	CGC	0.77	0.78	0.75	0.78	0.77	1.40	**1.31**	1.1	1.21
	CGG	0.90	0.88	0.89	0.91	0.91	0.50	1.29	1.21	1.25
	CGT	0.75	0.72	0.73	0.76	0.77	0.60	0.44	0.48	0.44
Ser	AGC	***2.20***	***2.22***	***2.07***	***2.18***	***2.26***	***2.10***	***1.62***	**1.44**	**1.49**
	AGT	0.62	0.61	0.62	0.6	0.63	0.40	0.77	0.9	0.85
	TCA	0.19	0.2	0.21	0.1	0.21	0.35	0.73	0.9	0.77
	TCC	1.24	1.18	1.24	1.31	1.24	1.60	1.5	1.31	1.45
	TCG	0.92	0.98	1.03	1.01	0.83	0.40	0.39	0.33	0.36
	TCT	0.82	0.81	0.83	0.8	0.83	1.15	0.99	1.13	1.08
Thr	ACA	1.04	1.04	1.04	1.04	1.05	1.03	0.92	1.14	1.03
	ACC	**1.40**	**1.32**	**1.42**	**1.39**	**1.42**	***2.22***	***1.68***	**1.42**	**1.55**
	ACG	0.44	0.46	0.42	0.44	0.43	0.27	0.57	0.46	0.52
	ACT	1.13	1.18	1.13	1.13	1.1	0.49	0.83	0.99	0.89
Val	GTA	0.62	0.63	0.63	0.62	0.61	0.23	0.34	0.47	0.41
	GTC	0.44	0.39	0.38	0.44	0.49	1.40	1.07	0.95	1.07
	GTG	0.74	0.74	0.75	0.75	0.73	***1.72***	***2.03***	***1.85***	***1.94***
	GTT	***2.21***	***2.24***	***2.25***	***2.2***	***2.17***	0.65	0.57	0.73	0.58
Tyr	TAC	0.78	0.86	0.77	0.8	0.76	**1.58**	**1.27**	**1.11**	**1.21**
	TAT	**1.22**	**1.14**	**1.23**	**1.2**	**1.24**	0.42	0.73	0.89	0.79

Notes: optimal codons are displayed in bold. Over-represented (RSCU > 1.6) codons are marked in bold and italics.

### The effect of mutation pressure and natural selection on codon usage bias

**Figure 3.**
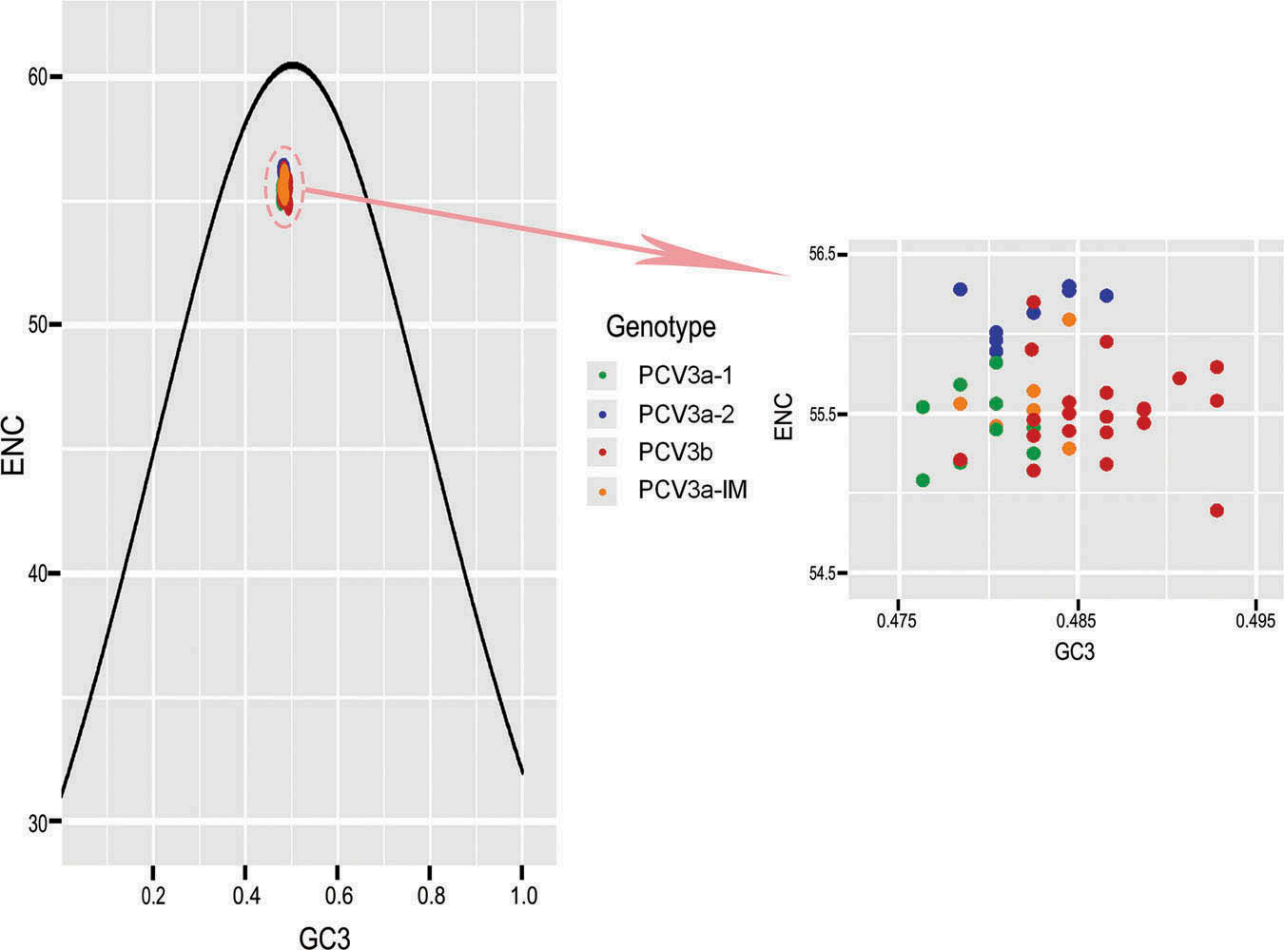
ENC-plot analysis (ENC plotted against GC_3s_). The black curve represents the expected curve derived from the positions of strains when the codon usage was only determined by the GC3s composition. PCV3a-1, PCV3a-2, PCV3a-IM and PCV3b are represented in green, blue, red and orange, respectively.

**Figure 4.**
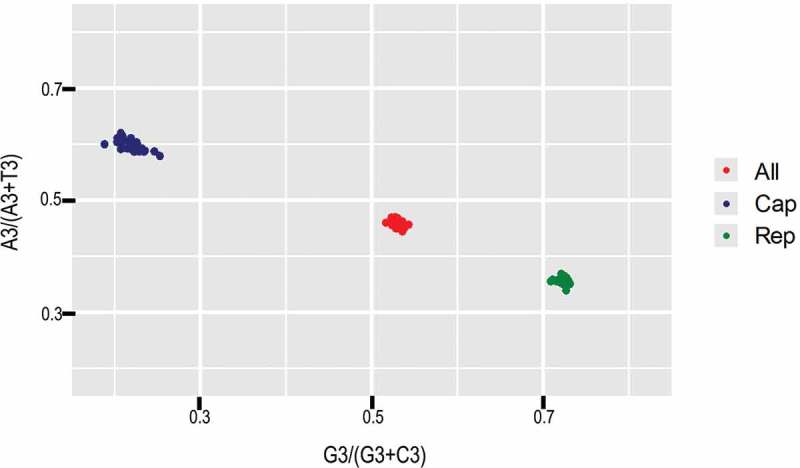
PR2 analysis of PCV3 and specific genes. Red, green and blue refer to complete coding sequences, ORF1 and ORF2, respectively.

To investigate the forces influencing the codon usage bias of PCV3, ENC-plot analysis of the different genotypes was carried out (Figure 3). We found that all strains sat below the standard curve regardless of genotype. Additionally, there was also a clear separation of different genotypes except for PCV3a-IM, showing that both mutation pressure and natural selection affect the codon usage bias of different genotypes. Moreover, we carried out correlation analysis of nucleotide composition, ENC, axis1, axis2, Aroma and Gravy (Table 2). A significant correlation was found between Gravy and ENC and GC_3s_ (r = −0.851, p < 0.01; r = 0.417, p < 0.01, respectively). Most of the parameters in the correlation analysis were related with each other, while Aroma only correlated with Gravy. In addition, PR2 analysis revealed that GC was used more frequently than AT (Figure 4). Overall, we found that both mutation pressure and natural selection influence the codon usage bias of PCV3.

**Table 2.** Correlation analysis among codon composition, ENC value, nucleic acid composition, Gravy, Aroma and axis 1, axis2.

	A%	C%	G%	T%	T3s	C3s	A3s	G3s	GC	GC3s	ENC	Gravy	Aromo	axis1
C%	−0.149													
G%	−0.733**	−0.362**												
T%	−0.071	−0.801**	0.170											
T3s	−0.174	−0.765**	0.333*	0.873**										
C3s	−0.336*	0.677**	−0.108	−0.436**	−0.316*									
A3s	0.833**	0.077	−0.682**	−0.256	−0.494**	−0.432**								
G3s	−0.764**	−0.221	0.881**	0.147	0.362**	−0.022	−0.788**							
GC	−0.731**	0.668**	0.452**	−0.627**	−0.465**	0.567**	−0.475**	0.491**						
GC3s	−0.796**	0.441**	0.515**	−0.312*	−0.102	0.739**	−0.810**	0.641**	0.837**					
ENC	0.079	0.563**	−0.358**	−0.461**	−0.503**	0.070	0.324*	−0.182	0.248	−0.012				
Gravy	−0.426**	−0.263	0.356*	0.424**	0.418**	0.320*	−0.620**	0.343*	0.046	0.417**	−0.366**			
Aromo	0.078	0.165	−0.042	−0.267	−0.051	−0.079	0.098	0.006	0.115	−0.047	0.105	−0.627**		
axis1	−0.469**	−0.279*	0.514**	0.321*	0.624**	0.291*	−0.777**	0.530**	0.144	0.495**	−0.485**	0.491**	0.208	
axis2	0.461**	−0.615**	−0.107	0.461**	0.311*	−0.388**	0.267	−0.242	−0.676**	−0.514**	−0.520**	−0.074	0.013	0.000

The **p < 0.01, * 0.05 < p < 0.01.

### Natural selection is the major force influencing the codon usage bias of PCV3

**Figure 5.**
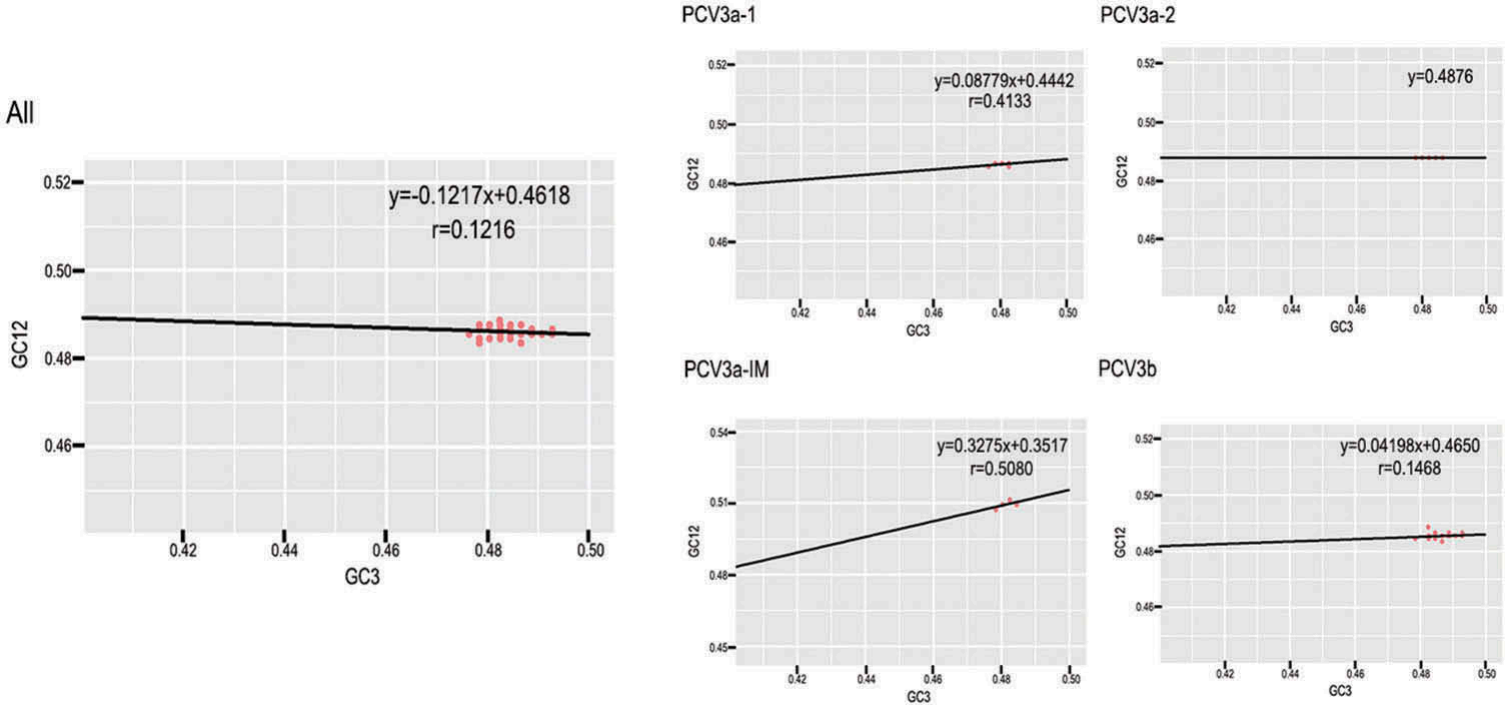
Neutrality plot analysis (GC_12s_ plotted against GC_3s_) for all the coding sequences of PCV3 and the different genotypes.

To understand which force between mutation pressure and natural selection had a bigger role driving codon usage bias, we performed neutrality analysis of all the sequences and grouped by genotype (Figure 5). The slope of the linear regression was −0.1217 for all the sequences, illustrating that mutation pressure accounted for 12.17% of the selection force while natural selection accounted for 87.83%. Additionally, the slopes of the linear regression among different genotypes were 8.78%, 0%, 32.75% and 4.2% for PCV3a-1, PCV3a-2, PCV3a-IM and PCV3b, respectively. Interestingly, mutation pressure had no effect on the codon usage bias of PCV3a-2. In summary, natural selection was the dominant role driving the codon usage bias of PCV3.

### PCV3 adaptation to host species

**Figure 6.**
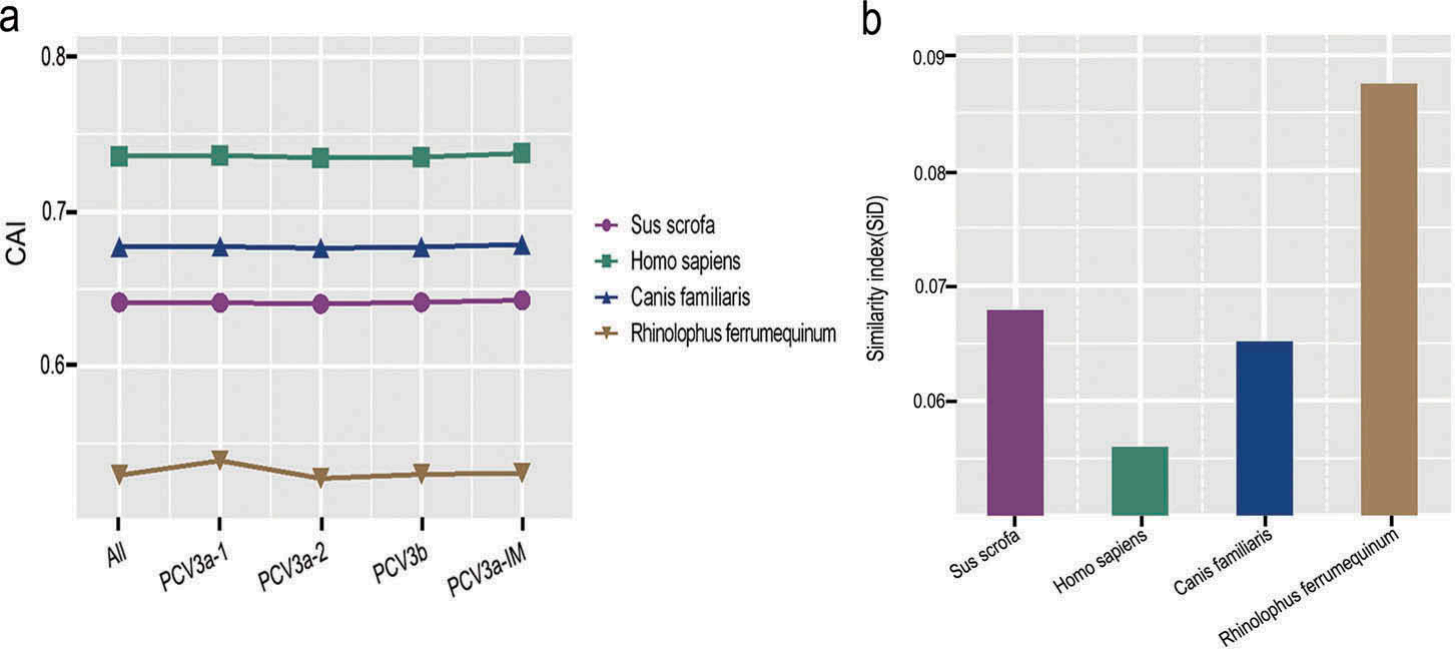
(a) CAI and (b) SiD analysis of different genotypes of PCV3 coding sequences in relation to potential host species, including *Sus scrofa* (purple), *Homo sapiens* (green), *Canis familiaris* (blue) and *Rhinclophus ferrumequinum* (brown).

Given that a recent analysis reported PCV3 in dogs [37], additionally, Wu et al. [38] and our previous study [36] discovered that PCV3 was found to be closely related to bat circovirus in China, we chose *Canis familiaris* and *Rhinolophus ferrumequinum* for analysis, especially since Rhinolophus spp. acts as a major reservoir for diverse mammalian viruses in China [39]. We used CAI and RCDI analysis to evaluate host suitability to PCV3. There were significant differences in CAI values among different host species (*Sus scrofa, Homo sapiens, Canis familiaris, Rhinolophus ferrumequinum*) (Figure 6(a)). In particular, *Homo sapiens* had the CAI value similar to *Sus scrofa*, and *Canis familiaris*, with a mean value of 0.7358 ± 0.002, while *Rhinolophus ferrumequinum* had the lowest with a mean value of 0.5296 ± 0.002 in the analysis of both, all the sequences and different genotypes. On the other hand, the mean RCDI values were 1.34 ± 0.01, 1.25 ± 0.01, 1.31 ± 0.01 and 1.59 ± 0.02 for *Sus scrofa, Homo sapiens, Canis familiaris* and *Rhinolophus ferrumequinum*, respectively, regardless of genotypes (Fig S2). This indicates that the highest codon deoptimization of PCV3 was towards *Rhinolophus ferrumequinum*. A similar trend was identified in the analysis of different genotypes. Interestingly, except for the high RCDI value of *Rhinolophus ferrumequinum*in in relation to PCV3a-2, the other host species exhibited low codon usage deoptimization. On the other hand, PCV3a-1 had the highest codon usage deoptimization compared to the other genotypes among all hosts apart from PCV3a-2 towards *Rhinolophus ferrumequinum*. Using SiD analysis, we found that *Rhinolophus ferrumequinum* had a significantly deeper effect on PCV3 coding sequences, followed by *Sus scrofa, Canis familiaris* and *Homo sapiens* (Figure 6(b)).

### Influence of dinucleotide frequencies on PCV3 codon usage bias

To detect the influence of dinucleotides in the codon usage pattern, the relative abundance of 16 dinucleotides was calculated (Fig S3). There were no under-represented dinucleotides (P_xy_ < 0.78), while 3 dinucleotides, including CpC, GpG and TpT, were over-represented (P_xy_ > 1.23). The RSCU value of these 3 dinucleotides CpC (GCC, CCA, CCC, CCG, CCT, TCC, ACC), GpG (GGA, GGC, GGG, GGT, AGG, CGG) and TpT (TTC, TTT, ATT, CTT, TTA, TTG, GTT) had 10 optimal synonymous codons among 18 optimal codons. Therefore, we can conclude that dinucleotides have an influence on the codon usage bias of PCV3.

## Discussion

PCV3, a novel emerging infectious virus, was firstly identified in the USA in 2015 [6,8] then mainly prevailing in China [38,40–43], South Korea [16], Brazil [17], Thailand [10] and European countries, such as Italy, Germany, Denmark, Spain, Sweden, Poland [9,11–15]. Until now, there has been no systematic codon analysis to understand its evolutionary history and codon usage patterns. In this study, we performed a codon usage analysis according to different genotypes and potential host species. As a newly evolved virus, PVC3 detection and epidemiological monitoring are not complete. Therefore, epidemiological investigation, real-time disease monitoring and other measures to prevent PCV3 from spreading among pigs worldwide and to other mammals, is strongly recommended. To date, the most effective method to inhibit transmission is via vaccination. The understanding of codon usage patterns may provide important clues to develop new and appropriate vaccines, therefore, the importance of this kind of studies [44].

Among the 52 strains, the China/GD2016 (KY418606) strain was not included in this study due to low quality and possibly misleading of the tree topology. Two stable clades: PCV3a and PCV3b were observed which was supported by phylogenetic and PCA analysis, reinforcing the fact that PCA can reflect genotypic classification based on evolutionary analysis [45]. Here, we found that A/G were abundant in coding sequences. Optimal synonymous codons ending in A/T were more abundant than G/C-ended codons. Altogether, this could indicate the existence of codon bias. However, we detected a high ENC value indicating low codon usage bias. Low codon usage bias has also been observed in other PCV strains, such as PCV1 (51.36) and PCV2 (54.31) [33] and other DNA viruses, including hepatitis B virus (56.31) [46] and iridovirus (range from 35.87 to 51.81) [47]. PCV3 had a lower codon bias than the other two porcine circoviruses. This might be due to the need of the virus to accommodate to the host replication system to replicate efficiency[48]. In this case, the low codon usage bias observed in PCV3 might be necessary to adapt to the natural host, pig, to prevail globally. However, this needs to be confirmed.

Although ENC values indicate the degree of codon preference, they do not provide insight into the factors contributing to codon usage bias. ENC-plots and correlation analysis revealed that both mutation pressure and natural selection, among other possible factors, contribute to the codon usage pattern of PCV3. Using neutrality analysis, we found that natural selection constrained the codon usage bias by 87.83% compared to mutation pressure (12.17%) using all PCV3 sequences. When the analysis was performed according to genotype, we found that the influence of natural selection on PCV3a-IM (67.25%) was slightly lower than the other genotypes. The reason for this is not clear, however we hypothesize that it could be due to the instable distribution of PCV3a-IM within the phylogeny. Overall, we found that natural selection was the dominant force driving the codon usage of PCV3.

We also found that dinucleotides influence the evolution of PCV3. There were no under-represented dinucleotides in PCV3 while the relative abundance of CpC, GpG and TpT deviated from the normal data and were over-represented. Based on RSCU analysis, synonymous codons harbouring these three dinucleotides occupied most of the prefer used codons, which means dinucleotide compositions played an important role in determining the patterns of codon usage of PCV3, apart from mutation pressure and natural selection. Although CpG was not under-represented, its content was low, which could be associated with the immunostimulatory nature of unmethylated CpGs. The recognition of unmethylated CpG by Toll-like receptor 9 leads to the activation of immune responses and thus, a low CpG content could be beneficial for virus replication [49].

For many viruses, the AT and GC contents are mostly related to the RSCU. We found that T-ended codons were more abundant compared to A/G/C-ended codons. Additionally, there was no difference in the usage of the 18 optimal codons among the different genotypes. However, A was the most abundant nucleotide. It has been suggested that the choice of optimal codons in viruses largely depends on the host [50]. PCV3 exhibited coincident and antagonistic codon usage patterns relative to its host when we contrasted the RSCU pattern of PCV3 to the host species, in agreement with other viruses such as hepatitis A virus [51]. This observed mixed codon usage pattern could be explained by the fact that coincident codons between virus and host are beneficial due to efficient protein translation, while antagonistic codons proper viral protein folding [52]. However, this speculation needs to be further confirmed.

To understand the relationship between virus and hosts further, we performed CAI, RCDI and SiD analysis. PCV3 was reported to close related with Chinese bat CVs by Wu et al .[38]. and our previous study [36]. Thus, we hypothesized that PCV3 may have evolved from bats and then gradually adapted to both pigs and dogs. CAI analysis revealed that, in comparison with other potential hosts, PCV3 displayed lowest CAI value in *Rhinolophus ferrumequinum*, while similar in *Sus scrofa, Homo sapiens* and *Canis familiars*, which was consistent with RCDI analysis. whereas, in contract to SiD analysis, indicating PCV3 developed strong tie with *Rhinolophus ferrumequinum* (as a origin), additionally, given previously reported that PCV3 has been detected in dogs [37], as well as the natural host swine, and PCVs related to the xenotransplants and vaccine contaminations [53,54], we hypothesized that potential cross-species transmission of PCV3, and might be risky to public health. Though, the infection of PCVs in human cells and pathogenicity of PCVs to public health was unclear [55], which still need further experimental research.

In conclusion, this study showed that the codon usage pattern of PCV3 coding sequences was affected by the interplay of different factors, such as mutation pressure, natural selection and dinucleotide compositions. The degree of codon usage preference was low and dominated mainly by natural selection. We also found evidence supporting the idea that PCV3 might be a potential threat to public health. Importantly, it has been reported that PCV3 infects dogs [37], increasing the potential risk of cross interspecies transmission and adding exposure of humans, though, currently, with unclear pathogenicity in human host. The findings of this study help us understand the underlying factors associated with PCV3 evolution and host adaption which will greatly serve future PCV3 research.

## Materials and methods

### Sequence data

A total of 52 completed genomes of PCV3 available until the 9^th^ of November 2017 were retrieved from the GenBank database (https://www.ncbi.nlm.nih.gov/genbank/) and considering the opposite direction of ORF2, the individual ORF1 and ORF2 gene were concatenated for the analysis. The detailed information of each strain including the accession number, strain name, country and collection-date are listed in Table S1.

### Recombination and phylogenetic analysis

Before the analysis, potential recombination events were examined by the recombination detection program (RDP4, version 4.39) [56]. Except for the method of LARD [57], other methods, including RDP [58], GENECONV [59], Chimaera [60], MaxChi [61], BootScan [62], SiSican [63] and 3Seq [64] were implemented to detect recombination events. The p value was set to 0.05. If at least four of the six methods detected recombination, the signal was considered to be recombination. Additionally, Bonferroni correction was applied to the analysis. Then in the phylogenetic analysis, sequences were aligned using ClustalW [65]. A pairwise distance matrix was calculated and clustered by the neighbor joining (NJ) method and that the statistical support of NJ tree was calculated by 1,000 bootstrap replicates which was reconstructed using MEGA 7.0 [65].

### Codon usage bias analysis

#### Nucleotide composition

Five nonsynonymous codons, including ATG, TGG and termination codons were excluded from the analysis. The frequencies of each nucleotide (A%, T%, C%, G%) and the total content of AT and GC were calculated using BioEdit (v7.0.9) [66]. The GC contents at the first, second and third positions (GC_1s_, GC_2s_, GC_3s_) were computed using EMBOSS: cusp (http://emboss.toulouse.inra.fr/cgi-bin/emboss/cusp). Additionally, the nucleotides at the third position of synonymous codons (A_3_%, T_3_%, C_3_% and G_3_%) were calculated using CodonW (v1.4.2) (http://codonw.sourceforge.net/culong.html#CodonW).

#### Effective number of codons (ENC)

The ENC value is used to detect the degree of codon usage bias. The value ranges from 20 to 61 [67]. The larger the value of ENC indicates a lower degree of preference. A value less than 35 indicates that the bias is significant and vice versa [68]. The ENC value was calculated by CodonW (v1.4.2) as follows:

$$ENC=2+\frac{9}{{\overset{ˉ}{F}}_{2}}+\frac{1}{{\overset{ˉ}{F}}_{3}}+\frac{5}{{\overset{ˉ}{F}}_{4}}+\frac{3}{{\overset{ˉ}{F}}_{6}}$$

where F_i_ (i = 2,3,4,6) is the mean of F_i_ for i-fold degenerate codon families. The F_i_ value was calculated as follows:

$$F_{i}=\frac{\overset{i}{\underset{j=1}{\sum }}{\left(\frac{n_{j}}{n}\right)}^{2}-1}{n-1}$$

where n is the total number of occurrences of the codons for that amino acid and n_j_ is the total number of occurrences of the j_th_ codon for that amino acid.

#### Principal component analysis (PCA)

As a multivariate statistical method, PCA is normally applied to study the relationship among variables and samples, which transform relative indices into small number of uncorrelated indices, thus, the so called principal components. In this study, each dimension represents a sense codon relative synonymous codon usage (RSCU) value [69]. PCA analysis was performed using GraphPad Prism 5.0.

#### Relative synonymous codon usage (RSCU)

The RSCU represents the usage frequencies of synonymous codons in amino acids excluding the effect of nucleotide composition and sequence length [70]. The RSCU value was calculated as follows:

$$RSCU=\frac{x_{ij}\;xn_{i.j}}{\overset{ni}{\underset{j}{\sum }}x_{ij}}$$

where $X_{ij}$ is the number of occurrence of the i_th_ codon for the j_th_ amino acid and n_i_ is the number of synonymous codons that encode the j_th_ amino acid [71] which was implemented in CodonW (v1.4.2). RSCU values > 1.0 and < 1.0 represent positive codon usage bias and negative codon usage bias, respectively [72]. In addition, a value < 0.6 indicates ‘underrepresented’ while > 1.6 indicates ‘over-represented’ [73].

### The effect of mutation pressure and natural selection on codon usage bias

#### Enc-plot analysis

ENC-plots (ENC value against GC_3s_ value) are used to reveal the factors driving codon usage bias. If mutation pressure is the only factor, the point will lie on the standard curve. Expected ENC values were calculated using the following formula:

$$ENC_{expected}=2+s+\frac{29}{\left(s^{2}+1-s^{2}\right)}$$

where ‘s’ refer to the frequency of G + C at the third codon position of synonymous codons.

#### Parity rule 2 (PR2) analysis

PR2 analysis was used to measure the effect of natural selection and mutation pressure. The [A_3_/(A_3_+ T_3_)] value is plotted in the ordinate while the [G_3_/(G_3_+ C_3_)] value in the abscissa. The origin is (x = 0.5 and y = 0.5), which indicates that there is no deviation between nucleotides A and G. Points sitting in the centre of the plot indicate equal roles of mutation pressure and natural selection [74,75].

#### Neutrality analysis

Neutrality analysis (GC_12s_ against GC_3s_) is used to determine which is the dominant factor affecting codon usage bias, and the neutrality plot was completed in GraphPad Prism 5.0. If the correlation line is close to the diagonal (high correlation) it indicates that external factors have little impact on codon usage bias, for example mutation pressure [50]. Similarity, if the correlation coefficient is towards the X or Y axis, natural selection is the dominant force [76].

### Analysis of host-specific adaptation

#### Codon adaptation index (CAI) analysis

The CAI values can estimate the degree of preference for codon usage of a gene based on the sequence of a known highly expressed gene. CAI values were calculated by the CAIcal SERVER (http://genomes.urv.cat/CAIcal/RCDI/) [77]. The reference database of the synonymous codon usage patterns (*Sus scrofa, Homo sapiens, Canis familiaris, Rhinolophus ferrumequinum*) was obtained from the Codon Usage Database (CUD) (http://www.kazusa.or.jp/codon/) [78]. CAI values range from 0 to 1. The higher the CAI value is indicative of stronger adaptability to the host [79].

#### Relative codon deoptimization index (RCDI) analysis

The RCDI of the different genotypes of PCV3 was calculated by the RCDI/eRCDI SERVER [80] (http://genomes.urv.cat/CAIcal/RCDI/) to show the codon usage deoptimization trend. A RCDI value of 1 indicates that the virus is predominantly adapted to the host, while a value higher than 1 indicates less adaptability [81]. The reference database was the same used for CAI analysis.

#### Similarity index (SiD) analysis

SiD was used to measure the effect of the host codon usage bias on PCV3. SiD was calculated as follows:

$$R\left(A,B\right)=\frac{\overset{59}{\underset{i=1}{\sum }}a_{i}xb_{i}}{\sqrt{\overset{59}{\underset{i=1}{\sum }}a_{i}^{2}x\overset{59}{\underset{i=1}{\sum }}b_{i}^{2}}}$$

$$D\left(A,B\right)=\frac{1-R\left(A,B\right)}{2}$$

where a_i_ means the RSCU value of 59 synonymous codons of the PCV3 coding sequences, b_i_ means the RSCU value of the identical codons of the potential host. The SiD value ranges from 0 to 1 [82]. The higher value indicates that the host has a dominant effect on the usage of codons.

### Codon dinucleotide frequency analysis

The dinucleotide frequencies were calculated using the DAMBE (v5.3.19) (http://dambe.bio.uottawa.ca/DAMBE/dambe.aspx) [83] software. The abundance and absence of dinucleotides in 16 dinucleotides with P_xy_ > 1.23, P_xy_ < 0.78 were analysed [84]. In addition, to understand if the dinucleotide composition plays a role in determining the codon usage pattern, the relationship between CpG and RSCU was analysed. The ratio value was calculated as follows:

$$P_{xy}=\frac{r_{xy}}{r_{x}r_{y}}$$

where r_x_ means the frequency of nucleotide X, r_y_ and r_xy_ are similar.

### Gravy and aroma statistics

The Gravy value is the mean of the sum of the hydropathic indices of each amino acid [85] which indicates the effect of protein hydrophobicity on codon usage bias calculated by CodonW (v1.4.2). The value ranges from −2 to 2. The Aroma value measures the effect of aromatic hydrocarbon proteins on codon usage bias.

### Statistical analysis

The correlations among the A%, T%, G%, C%, A_3s,_ T_3s_, G_3s_, C_3s_, GC_3s_, ENC, Aroma and Gravy were calculated using Graphpad Prism 5.0, with an extremely significant relationship (**) of p < 0.01 and a significant relationship (*) of 0.01 < p < 0.05.
