# Supplementary material for: Insights into the genetic and host adaptability of emerging porcine circovirus 3
Source: Virulence. 2018 Aug 24;9(1):1301–13. doi: 10.1080/21505594.2018.1492863 (PMC6177243; doi:10.1080/21505594.2018.1492863)
Supplement: Supplemental Material [file kvir-09-01-1492863-s001.pdf]

Supporting Information:

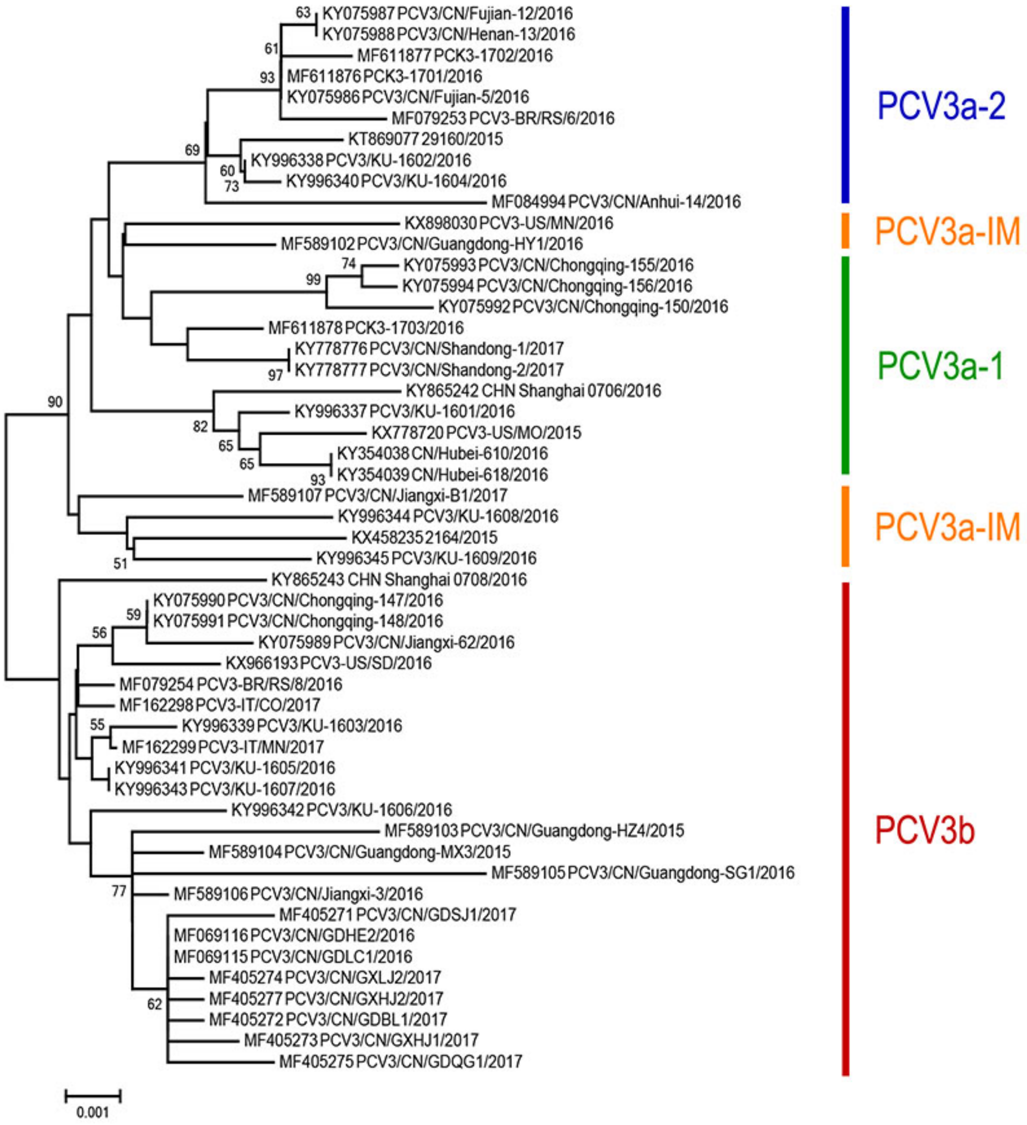

Fig S1. Phylogenetic tree constructed with 51 complete coding sequences (main known coding regions). NJ tree of PCV3 coding sequences. Bootstraps higher than 50 are displayed along the relative branches. PCV3a-1, PCV3a-2, PCV3a-IM and PCV3b are represented in green, blue, red and orange, respectively.

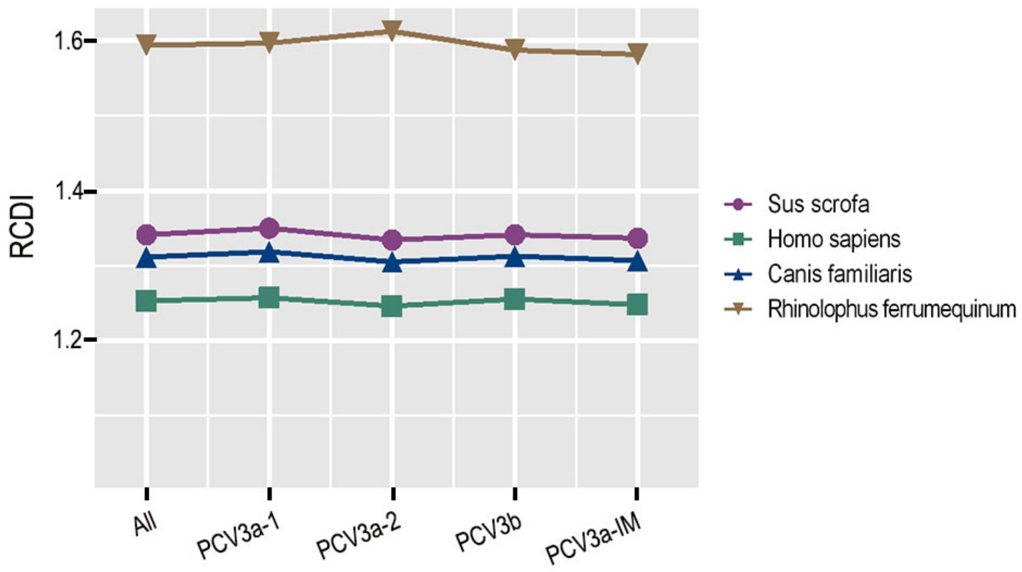

Fig S2. RCDI analysis of different genotypes of PCV3 coding sequences in relation to potential host species including *Sus scrofa* (purple), *Homo sapiens* (green), *Canis familiaris* (blue) and *Rhinclophus ferrumequinum* (brown).

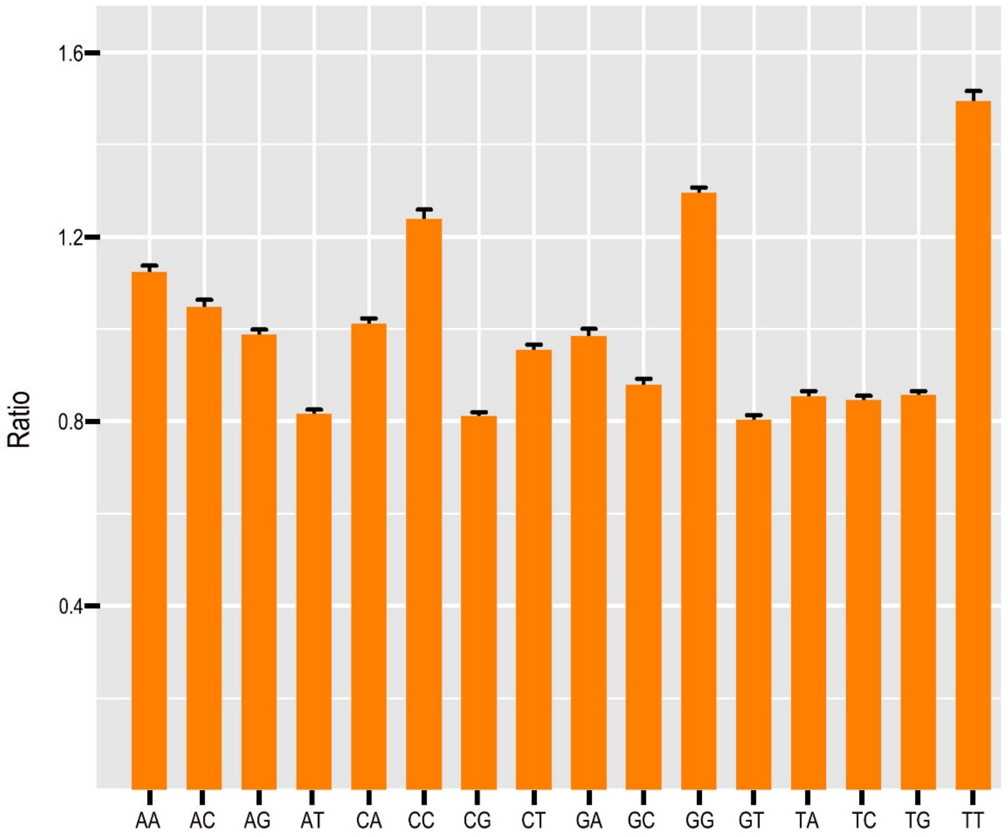

Fig S3. The frequency of 16 dinucleotides.

Table S1. The detailed information of each strain including the accession number, strain name , country and collection-date of PCV3 analyzed in this research.

Accession number	Strain	Country	Host	Year
MF069115	PCV3/CN/GDLC1/2016	China	swine	2016
MF069116	PCV3/CN/GDHE2/2016	China	swine	2016
MF405271	PCV3/CN/GDSJ1/2017	China	swine	2017
MF405272	PCV3/CN/GDBL1/2017	China	swine	2017
MF405273	PCV3/CN/GXHJ1/2017	China	swine	2017
MF405274	PCV3/CN/GXLJ2/2017	China	swine	2017
MF405275	PCV3/CN/GDQG1/2017	China	swine	2017
MF405276	PCV3/CN/GXLJ1/2017	China	swine	2017
KT869077	29160	USA: North Carolina	swine	2015
KX458235	2164	USA: Oklahoma	swine	2015
KX778720	PCV3-US/MO2015	USA	swine	2015
KX898030	PCV3-US/MN2016	USA	swine	2016
KX966193	PCV3-US/SD2016	USA	swine	2016
KY075986	PCV3/CN/Fujian-5/2016	China	swine	2016

KY075987	PCV3/CN/Fujian-12/2016	China	swine	2016
KY075988	PCV3/CN/Henan-13/2016	China	swine	2016
KY075989	PCV3/CN/Jiangxi-62/2016	China	swine	2016
KY075990	PCV3/CN/Chongqing-147/2016	China	swine	2016
KY075991	PCV3/CN/Chongqing-148/2016	China	swine	2016
KY075992	PCV3/CN/Chongqing-150/2016	China	swine	2016
KY075993	PCV3/CN/Chongqing-155/2016	China	swine	2016
KY075994	PCV3/CN/Chongqing-156/2016	China	swine	2016
KY354038	CN/Hubei-610/2016	China	swine	2016
KY354039	CN/Hubei-618/2016	China	swine	2016
KY778776	PCV3/CN/Shandong-1/201703	China	swine	2017
KY778777	PCV3/CN/Shandong-2/201703	China	swine	2017
KY865242	CHN_Shanghai_0706_2016	China	swine	2016
KY865243	CHN_Shanghai_0708_2016	China	swine	2016
KY996337	PCV3/KU-1601	South Korea	swine	2016
KY996338	PCV3/KU-1602	South Korea	swine	2016
KY996339	PCV3/KU-1603	South Korea	swine	2016
KY996340	PCV3/KU-1604	South Korea	swine	2016
KY996341	PCV3/KU-1605	South Korea	swine	2016
KY996342	PCV3/KU-1606	South Korea	swine	2016
KY996343	PCV3/KU-1607	South Korea	swine	2016
KY996344	PCV3/KU-1608	South Korea	swine	2016
KY996345	PCV3/KU-1609	South Korea	swine	2016
MF079253	PCV3-BR/RS/6	Brazil	swine	2016
MF079254	PCV3-BR/RS/8	Brazil	swine	2016
MF084994	PCV3/CN/Anhui-14/201611	China	swine	2016
MF162298	PCV3-IT/CO2017	Italy	swine	2017
MF162299	PCV3-IT/MN2017	Italy	swine	2017
MF589102	PCV3/CN/Guangdong-HY1/2016	China	swine	2016
MF589103	PCV3/CN/Guangdong-HZ4/2015	China	swine	2015
MF589104	PCV3/CN/Guangdong-MX3/2015	China	swine	2015
MF589105	PCV3/CN/Guangdong-SG1/2016	China	swine	2016
MF589106	PCV3/CN/Jiangxi-3/2016	China	swine	2016
MF589107	PCV3/CN/Jiangxi-B1/2017	China	swine	2017
MF611876	PCK3-1701	South Korea	swine	2016
MF611877	PCK3-1702	South Korea	swine	2016
MF611878	PCK3-1703	South Korea	swine	2016
MF405277	PCV3/CN/GXHJ2/2017	China	swine	2017

Table S2. The nucleotide compositions of PCV3 complete sequences, excluding non-synonymous and terminal codons.

Accession number	A%	C%	G%	T%	T_{3s}	C_{3s}	A_{3s}	G_{3s}	AT	GC	GC_{1s}	GC_{2s}	GC_{12s}	AT_{3s}
KY075992	28.52	22.5	25.98	23.02	0.3366	0.2902	0.298	0.3184	0.516	0.485	0.5072	0.466	0.4866	0.5196
KY075993	28.59	22.3	25.91	23.16	0.3415	0.2854	0.298	0.3193	0.518	0.483	0.5072	0.4639	0.4856	0.5237
KY075994	28.52	22.4	25.98	23.09	0.339	0.2854	0.298	0.3221	0.516	0.484	0.5093	0.4639	0.4866	0.5216
KY354038	28.38	22.3	26.19	23.09	0.3415	0.2854	0.291	0.3277	0.515	0.485	0.5093	0.4639	0.4866	0.5175
KY354039	28.38	22.3	26.19	23.09	0.3415	0.2854	0.291	0.3277	0.515	0.485	0.5093	0.4639	0.4866	0.5175
KY778776	28.38	22.5	25.91	23.16	0.3415	0.2878	0.293	0.3212	0.516	0.485	0.5093	0.4639	0.4866	0.5196
KY778777	28.38	22.5	25.91	23.16	0.3415	0.2878	0.293	0.3212	0.516	0.485	0.5093	0.4639	0.4866	0.5196
KY865242	28.25	22.3	26.19	23.3	0.3463	0.2829	0.285	0.3305	0.516	0.485	0.5072	0.4639	0.4856	0.5175
KY996337	28.45	22.1	26.12	23.3	0.3463	0.2805	0.293	0.3249	0.518	0.483	0.5093	0.4619	0.4856	0.5237
MF611878	28.52	22.4	26.05	23.02	0.3399	0.2861	0.295	0.324	0.516	0.485	0.5093	0.4639	0.4866	0.5196
KX778720	28.45	22.1	26.25	23.16	0.3463	0.278	0.291	0.3305	0.516	0.484	0.5093	0.4639	0.4866	0.5216
KY075987	28.32	22.7	26.05	22.96	0.3366	0.2902	0.291	0.3268	0.513	0.487	0.5113	0.4639	0.4876	0.5134
KY075986	28.38	22.7	25.98	22.96	0.3366	0.2902	0.293	0.324	0.513	0.487	0.5113	0.4639	0.4876	0.5155
KY075988	28.32	22.7	26.05	22.96	0.3366	0.2902	0.291	0.3268	0.513	0.487	0.5113	0.4639	0.4876	0.5134
KY996338	28.45	22.6	25.91	23.02	0.339	0.2878	0.296	0.3212	0.515	0.485	0.5113	0.4639	0.4876	0.5196
KY996340	28.52	22.6	25.91	22.96	0.3366	0.2878	0.298	0.3212	0.515	0.485	0.5113	0.4639	0.4876	0.5196
MF611876	28.38	22.7	25.98	22.96	0.3366	0.2902	0.293	0.324	0.513	0.487	0.5113	0.4639	0.4876	0.5155
MF611877	28.38	22.5	26.05	23.02	0.339	0.2878	0.293	0.324	0.514	0.486	0.5113	0.4639	0.4876	0.5175
MF084994	28.38	22.5	26.05	23.09	0.3415	0.2829	0.293	0.3268	0.515	0.485	0.5113	0.4639	0.4876	0.5196
MF079253	28.38	22.8	25.91	22.96	0.3358	0.292	0.294	0.3221	0.513	0.487	0.5113	0.4639	0.4876	0.5155
KT869077	28.45	22.5	25.91	23.09	0.339	0.2854	0.298	0.3212	0.516	0.485	0.5113	0.4639	0.4876	0.5216
KY075990	28.32	22.4	26.12	23.16	0.3447	0.2885	0.285	0.3277	0.515	0.485	0.5113	0.4598	0.4856	0.5155
KY075991	28.32	22.4	26.12	23.16	0.3447	0.2885	0.285	0.3277	0.515	0.485	0.5113	0.4598	0.4856	0.5155
KY075989	28.32	22.2	26.19	23.3	0.3472	0.2836	0.285	0.3305	0.516	0.484	0.5093	0.4598	0.4846	0.5175
KY865243	28.52	22.5	25.91	23.09	0.3423	0.291	0.291	0.323	0.516	0.484	0.5113	0.4577	0.4845	0.5175
KY996339	28.38	22.3	26.12	23.16	0.3464	0.2899	0.283	0.3305	0.516	0.485	0.5113	0.4557	0.4835	0.5134
KY996341	28.38	22.3	26.12	23.16	0.3456	0.2892	0.285	0.3277	0.516	0.485	0.5113	0.4577	0.4845	0.5155
KY996342	28.38	22.5	25.98	23.16	0.3464	0.2924	0.283	0.3277	0.516	0.485	0.5113	0.4557	0.4835	0.5134
KY996343	28.38	22.3	26.12	23.16	0.3456	0.2892	0.285	0.3277	0.516	0.485	0.5113	0.4577	0.4845	0.5155
MF405272	28.18	22.5	26.19	23.09	0.3423	0.2934	0.283	0.3277	0.513	0.487	0.5134	0.4598	0.4866	0.5113
MF069116	28.25	22.5	26.12	23.09	0.3423	0.2934	0.283	0.3277	0.513	0.487	0.5113	0.4598	0.4856	0.5113
MF069115	28.25	22.5	26.12	23.09	0.3423	0.2934	0.283	0.3277	0.513	0.487	0.5113	0.4598	0.4856	0.5113
MF405275	28.11	22.6	26.25	23.02	0.339	0.2951	0.28	0.3305	0.511	0.489	0.5113	0.4619	0.4866	0.5072

MF405271	28.18	22.5	26.25	23.02	0.3399	0.2934	0.28	0.3333	0.512	0.488	0.5113	0.4598	0.4856	0.5072
MF589103	28.59	22.5	25.77	23.16	0.3447	0.2934	0.293	0.3137	0.518	0.483	0.5093	0.4598	0.4846	0.5216
MF589104	28.38	22.5	25.98	23.16	0.3447	0.291	0.288	0.3221	0.516	0.485	0.5113	0.4598	0.4856	0.5175
MF589105	28.43	22.6	26.02	22.91	0.3407	0.2868	0.293	0.3258	0.514	0.487	0.5135	0.4638	0.4887	0.5176
MF405273	28.25	22.7	26.12	22.96	0.3374	0.2983	0.283	0.3277	0.512	0.488	0.5113	0.4598	0.4856	0.5072
MF405277	28.25	22.6	26.12	23.02	0.3399	0.2958	0.283	0.3277	0.513	0.487	0.5113	0.4598	0.4856	0.5093
MF405274	28.25	22.5	26.12	23.16	0.3447	0.291	0.283	0.3277	0.514	0.486	0.5113	0.4598	0.4856	0.5134
MF589106	28.32	22.6	25.98	23.09	0.3423	0.2934	0.285	0.3249	0.514	0.486	0.5113	0.4598	0.4856	0.5134
MF079254	28.32	22.3	26.12	23.23	0.3472	0.2861	0.285	0.3277	0.516	0.485	0.5113	0.4598	0.4856	0.5175
MF162298	28.25	22.4	26.19	23.16	0.3447	0.2885	0.285	0.3277	0.514	0.486	0.5134	0.4598	0.4866	0.5155
MF162299	28.25	22.4	26.19	23.16	0.3447	0.2885	0.283	0.3305	0.514	0.486	0.5113	0.4598	0.4856	0.5134
KX966193	28.11	22.4	26.25	23.23	0.3472	0.2885	0.278	0.3333	0.513	0.487	0.5113	0.4598	0.4856	0.5113
KX966193	28.32	22.4	26.19	23.09	0.3415	0.2878	0.291	0.3249	0.514	0.486	0.5113	0.4639	0.4876	0.5175
MF589102	28.32	22.5	26.05	23.09	0.3415	0.2878	0.291	0.3249	0.514	0.486	0.5113	0.4639	0.4876	0.5175
KX458235	28.45	22.3	25.84	23.37	0.3463	0.2878	0.291	0.3202	0.518	0.482	0.5072	0.4598	0.4835	0.5216
KY996344	28.52	22.6	25.91	22.96	0.3399	0.2934	0.291	0.3221	0.515	0.485	0.5093	0.4619	0.4856	0.5155
MF589107	28.38	22.5	25.98	23.09	0.3423	0.2885	0.288	0.3287	0.515	0.485	0.5093	0.4619	0.4856	0.5155
KY996345	28.59	22.4	25.91	23.09	0.3399	0.2885	0.296	0.3221	0.517	0.483	0.5093	0.4598	0.4846	0.5196
Average	28.31	22.5	26.09	23.13	0.3436	0.2906	0.285	0.327	0.514	0.486	0.5112	0.4598	0.4855	0.5143
SD	0.111	0.11	0.12	0.096	0.0027	0.0033	0.004	0.004	0.002	0.002	0.0012	0.002	0.0012	0.0039
